# Telephone outreach by volunteer navigators: a theory-based evaluation of an intervention to improve access to appropriate primary care

**DOI:** 10.1186/s12875-023-02096-4

**Published:** 2023-08-21

**Authors:** Jeannie Haggerty, Mélanie-Ann Smithman, Christine Beaulieu, Mylaine Breton, Émilie Dionne, Virginia Lewis

**Affiliations:** 1 Department of Family Medicine, McGill Research Chair in Family & Community Medicine at St. Mary’s, McGill University, St. Mary’s Research Centre, Montreal, Canada; 2 IMPACT Team, St. Mary’s Research Centre, 3830 Av. Jean-Brillant Ave, Pavillon Hayes, #4720, Montréal, Québec H3T 1M5 Canada; 3 St. Mary’s Research Centre, 3830 Av. Jean-Brillant Ave, Pavillon Hayes, Montréal, Québec H3T 1M5 Canada; 4 Université de Sherbrooke, Campus Longueuil, Centre de Recherche Charles-Le Moyne Sur Les Innovations en Santé, 150, Place Charles-Le Moyne C. P. 200, Longueuil, Québec J4K 0A8 Canada; 5 Faculté de Médecine Et Des Sciences de La Santé, Université de Sherbrooke, 3001 12 Ave N Immeuble X1, Sherbrooke, Québec J1H 5N4 Canada; 6 VITAM – Centre de recherche en santé durable, Université Laval, 2480, Chemin de La Canardière, Québec, Québec G1J 2G1 Canada; 7 Australian Institute for Primary Care & Ageing, La Trobe University, Melbourne, VIC 3086 Australia

**Keywords:** Canada, Action research, Access to primary health care, Patient-centered accessibility framework, Community health workers, Volunteer patient navigators

## Abstract

**Background:**

A pilot intervention in a participatory research programme in Québec, Canada, used telephone outreach by volunteer patient navigators to help unattached persons from deprived neighbourhoods attach successfully to a family doctor newly-assigned to them from a centralized waiting list. According to our theory-based program logic model we evaluated the extent to which the volunteer navigator outreach helped patients reach and engage with their newly-assigned primary care team, have a positive healthcare experience, develop an enduring doctor-patient relationship, and reduce forgone care and emergency room use.

**Method:**

For the mixed-method evaluation, indicators were developed for all domains in the logic model and measured in a telephone-administered patient survey at baseline and three months later to determine if there was a significant difference. Interviews with a subsample of 13 survey respondents explored the mechanisms and nuances of intended effects.

**Results:**

Five active volunteers provided the service to 108 persons, of whom 60 agreed to participate in the evaluation. All surveyed participants attended the first visit, where 90% attached successfully to the new doctor. Indicators of abilities to access healthcare increased statistically significantly as did ability to explain health needs to professionals. The telephone outreach predisposed patients to have a positive first visit and have trust in their new care team, establishing a basis for an enduring relationship. Patient-reported access difficulties, forgone care and use of hospital emergency rooms decreased dramatically after patients attached to their new doctors.

**Conclusions:**

As per the logic model, telephone outreach by volunteer navigators significantly increased patients’ abilities to seek, reach and engage with care and helped them attach successfully to newly-assigned family doctors. This light-touch intervention may have promise to achieve of the intended policy goals for the centralized waiting list to increase population access to appropriate primary care and reduce forgone care.

**Supplementary Information:**

The online version contains supplementary material available at 10.1186/s12875-023-02096-4.

## Background

Access to primary care services is a feature of high-performing health systems because most health concerns are managed in primary care and persons can be referred to other community and specialized services [1, 2]. While Canada’s publicly-funded medical system has addressed the issue of affordability of care, [3]. Canada has performed poorly compared to peer countries on access to a regular primary care provider and timeliness of care  [4]. Enhancing access to primary healthcare has been a policy priority in Canada [5, 6].

Access to comprehensive and appropriate primary care in Canada’s publicly-funded health system depends on being attached to a most responsible primary care doctor. Unattached persons are at significant risk of experiencing forgone care, [7] emergency room use [8–10] and less comprehensive primary care [9, 11]. Within Canada, the province of Québec (population 8 million) has the highest rate of unattached persons, hovering at approximately 25% for the last two decades [12]. The policy response in 2008 was to create centralized waiting lists in each local health network from which family doctors could select new patients for their practice. However, the centralized waiting list underrepresents persons from socially-or-materially deprived neighbourhoods compared to the general population, [13] and our analysis of regional health administrative data showed that persons from deprived neighbourhoods waited 3–5 times longer than others on the list. Furthermore, persons from deprived neighbourhoods are also more likely to be put back on the waiting list for contact failures or because the patient did not attend their first visit to the newly assigned family doctor.

This finding of inequitable access to the centralized waiting list is in keeping with evidence that health service programs and innovations tend to benefit the better-off more than the disadvantaged, [14–16]. Socially vulnerable patients – those who lack the social resources to withstand stressors inherent to organisations and systems – often experience gaps and barriers in accessing needed healthcare [17]. In order to achieve equity, interventions need to have a pro-vulnerable orientation [18].

Our objective is to evaluate the potential effectiveness of telephone outreach by volunteer navigators to persons from socially or materially deprived neighbourhoods who had been newly-assigned to a primary care doctor from the centralised waiting list. The intervention was guided by a theory-based program logic model which hypothesized that information and navigational support from peer navigator would help new patients reach and engage with their newly assigned care team, have a positive healthcare experience, develop an enduring doctor-patient relationship, and reduce forgone care and emergency room use. Before reporting on the extent to which the the intervention achieved the hypothesized outputs, impacts and outcomes, we first describe the setting then present and the volunteer navigator intervention then our theory-based logic model.

### Context and setting

This study was part of “Innovative Models Promoting Access-to-Care Transformation (IMPACT),” a 5-year (2013–2018) Canada-Australia action research programme to improve access to appropriate primary care for vulnerable populations. Action research identifies a local need, then works collaboratively with key stakeholders to design, implement and test an intervention through cycles of problem identification, study and reflection. The three Canadian provinces (Alberta, Ontario, Québec) and the two Australian states (New South Wales, Victoria) identified different access problems and vulnerable populations but shared a common theory-based logic model operational and evaluation approach. The theory-based evaluation across sites has been presented elsewhere [19].

In the Quebec study region of Montérégie, approximately 30% of persons assigned a new doctor were returned to the waiting list (personal communication, M. Décarie). Inspired by the community health worker model [20] the Quebec IMPACT partnership designed a peer patient navigation intervention to help persons attach to a newly assigned doctor from the centralized waiting list. Navigators provide general support to link patients to needed healthcare and are an increasingly popular strategy to reduce barriers to healthcare [21, 22]. A review suggests that navigators in primary care have the potential to reduce patient barriers to healthcare [23] partly because navigation information is better received and understood from a peer who is closer to the patient’s social reality than health professionals [24, 25]. The project coincided with a massive health system administrative restructure and budget cuts, and the volunteer navigator project was perceived by health system partners as a low-cost and potentially sustainable intervention.

### Volunteer navigator intervention

The names of new patients were sent to the volunteer coordinator at the same time they were sent to the family doctor’s clinic, and the coordinator selected those whose neighborhood postal code was classified in the two lowest quintiles of social or material deprivation [26]. The volunteer coordinator dispatched the information to available volunteer navigators. The implementation details are described elsewhere (Haggerty J, Beaulieu C, Smithman MA, Dionne É, Breton M: Volunteer Patient Navigators: considerations for feasibility and implementation of a promising intervention to attach materially-or-socially-deprived persons to primary care, forthcoming).

The intervention consisted of a single call, though volunteer navigators were free to make additional follow-up calls at their discretion or if requested by the new patients. Volunteers made up to 5 contact attempts at different times, including outside regular office hours, before the first visit. In the call, volunteers identified themselves as volunteers offering a ‘welcome service’ to patients assigned to a new family doctor at the clinic, and informed the person of the consequence of not attending the first visit. They then reminded the patient about documents required for the first visit, offered logistical information about how to travel to the clinic, affirmed that this was a medical visit and offered visit preparation tips. Finally, they emailed brochures with information about the clinic and how to prepare for medical visits. Calls lasted 10 min on average.

### Québec theory-based program logic and design

**Fig. 1:**
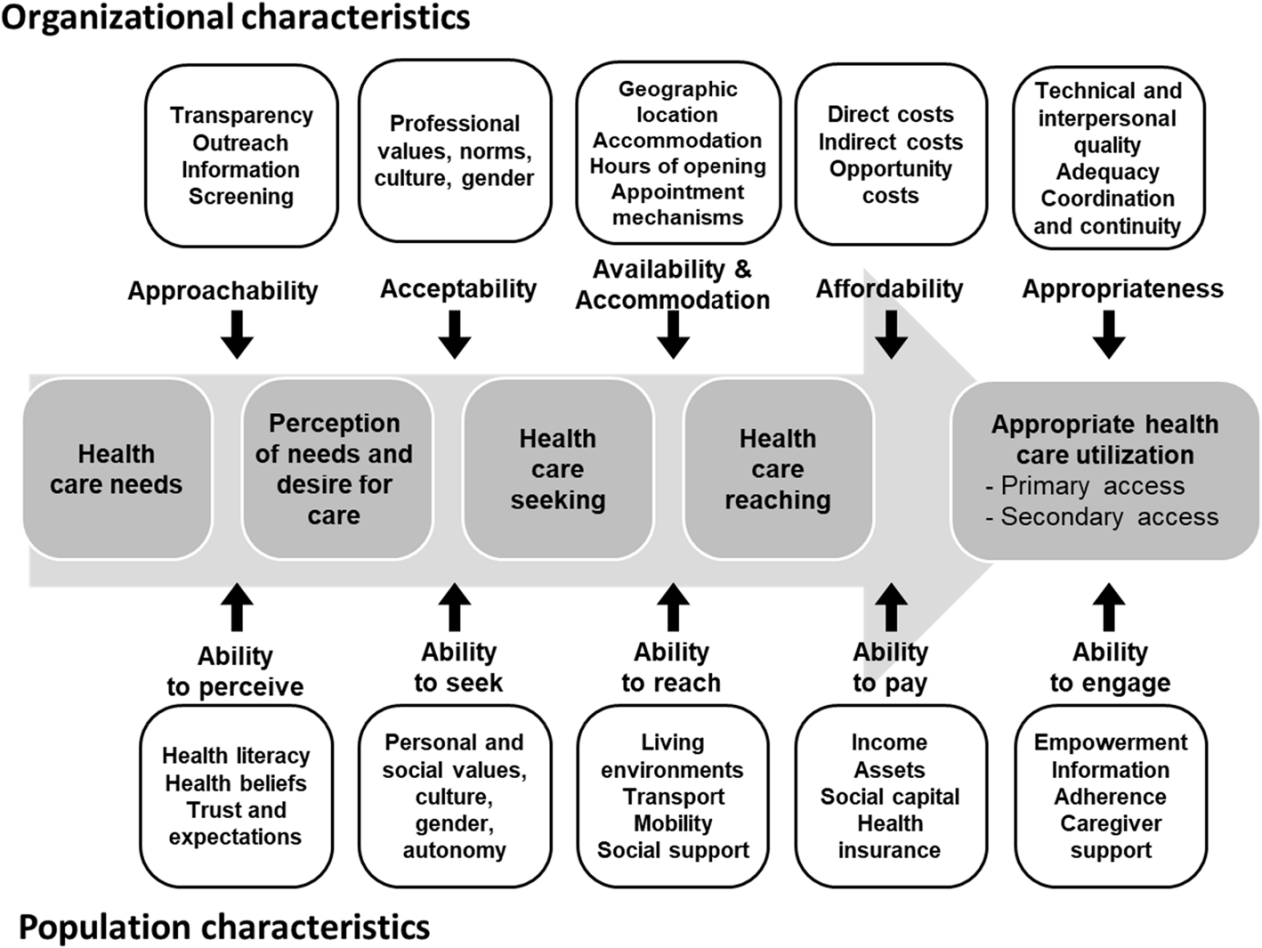
The Patient-Centered Accessibility Framework, showing organizational and patient characteristics leading to appropriate healthcare use (Adapted with author permission from Levesque, Harris, Russell. International Journal for Equity in Health 2013;12:18–27)

The Quebec theory-based logic model was designed as a sub-set of the full IMPACT logic model that pertained to a comprehensive IMPACT approach to increasing primary care access for vulnerable populations [19]. The principal theory base is the Patient-Centered Accessibility Framework, [27] which is a synthesis of existing conceptual models of access. It posits that patient abilities and organizational characteristics interact at different access stages on the pathway to obtaining appropriate care. The Framework is depicted in Fig. 1.

**Fig. 2:**
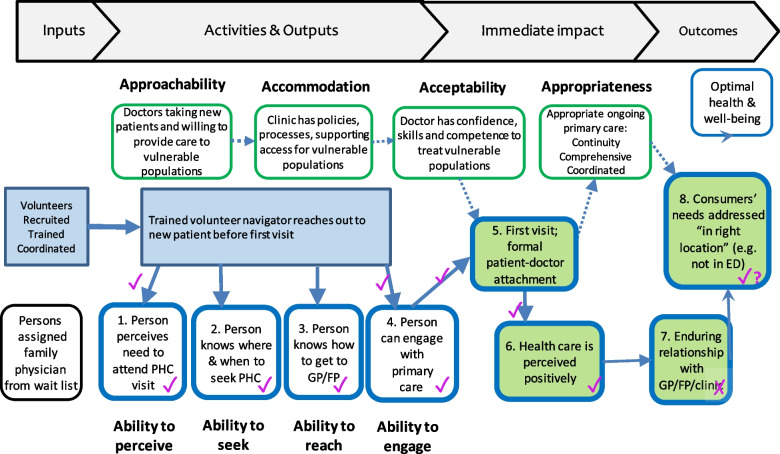
Theory-based program logic model for Québec volunteer navigator intervention. Legend: Intended outputs, impacts and outcomes shown in bold outline dimension boxes; hypothesized causal pathway arrows, bold = studied, dashed = presumed; evaluation that intervention confirms hypothesis (  ) or not (  )

The IMPACT approach implemented interventions to modify organizational accessibility characteristics (approachability, acceptability, availability & accommodation, affordability and appropriateness) to better respond to the ability of the vulnerable population to perceive, seek, reach, afford, and actively engage care. An initial 2016 intervention used clinic resources and social workers to reach out to newly-assigned patients from deprived neighbourhoods, but it was discontinued when the rapid-cycle evaluation showed that clinic staff could not increase contact attempts and that patient needs did not warrant social worker expertise. Consequently, we pivoted to the light-touch intervention with trained community volunteer navigators, achieving our intended impacts and outcomes by focusing exclusively on outputs on the population side of the Patient Centered Accessibility Framework, as represented in Fig. 2.

Arranged from left to right are the inputs (or starting conditions), the activities and outputs, followed by the immediate impacts and the ultimate outcomes. The bold solid arrows show the expected causal pathways at the patient level. Although there were no measured activities or outputs at the provider level (boxes outlined in green) the presumed casual pathways are shown as dashed arrows.

The telephone outreach by volunteer navigators is an activity at the start of the access trajectory. The expected outputs are that the patient: Box-1) perceives the importance of the first visit; Box-2) knows where and when to seek care at the new clinic (information pamphlet); Box-3) knows how to reach the clinic by usual means of transport, and; Box4) can engage in healthcare visit through visit preparation material. The anticipated immediate outcome (Box 5) is that patients attend the first visit prepared for a medical visit and become formally attached to the new family. The principal impact (Box 6) was that the first visit would be perceived positively and lead to successful attachment to the assigned family doctor. This was the first step to an intended outcome (Box 7) of an enduring therapeutic doctor-patient relationship.

The ultimate outcome (Box 8) of appropriate care was presumed to accrue through subsequent successful access to ongoing primary care, where the patient gets the right care in the right place in a timely manner (Box 10), as measured by increased referral rates to other services (especially community and social services) reduced rates of unmet needs for healthcare, reduced emergency room visits for minor problems.

## Methods

We used a mixed-method pre-post design to evaluate to the potential effectiveness of volunteer navigator intervention as proposed by our theory-based logic model. The pilot intervention took place between November 2017 and March 2018. All evaluation participants were adults (aged 18 + years) and able to respond verbally in English or French. No monetary incentive was offered to either patients or volunteers. The quantitative indicators for outputs (ability to seek, reach, and engage), primary impact (positive first visit) and intended outcomes (enduring relationship, appropriate care) were collected in a patient survey at baseline and 3-months later. Qualitative interviews were used to explore the causal pathways in a subset of survey respondents.

### Data collection and analysis

The common set of IMPACT quantitative indicators were determined for each of the domains in the logic model, using questions from validated instrument wherever possible. The patient survey was administered by telephone in either English or French by a research assistant (not the volunteer navigator) just after the volunteer navigator call and three months later.

The patient surveys elicited data about general healthcare use, reports of difficulties accessing care, forgone care or use of the emergency room, as well as confidence, trust and sense of being known by the provider most consulted over the previous 6 months at baseline and in the 3 after (follow-up). We used indicators from validated instruments wherever possible, though some were adapted for the IMPACT context. Most used evaluative Likert response options, but were adapted from a 5-point to a 4-point scale as more appropriate for telephone administration.

Qualitative semi-structured interviews with a subgroup of 13 survey respondents were conducted at baseline and three months after the initial call. In qualitative interviews participants described the volunteer navigator call they had received and how it had helped them. They also described their most recent healthcare experience, and if there had been any other changes in the previous 3 months. Interview question were framed to avoid implications of causality.

Additional data about the impact of the calls came from a single discussion group with the 5 active volunteer navigators toward the end of the pilot implementation (March 2018) by the volunteer coordinator. The group interview elicited volunteers’ impressions of how persons received the services and elements of success or failure, as well as exploring the motivations of and impacts on volunteer participation.

#### Quantitative analysis

Our principal analysis of potential effectiveness is the difference in patient reports between baseline and the three-month follow-up. For continuous or ordinal variables we used the paired t-test to determine if the average difference between baseline and follow-up is different from zero, using a two-tailed critical value for *p* < 0.05 for statistical significance. We also used the non-parametric Wilcoxon ranked to verify the robustness of the results. For comparison of paired binary events (or percentages), we used the McNemar test to assess whether the difference in distribution of events is statistically significant between baseline and follow-up. The number of paired observations varied by indicator because not all indicators at follow-up had counterparts during the baseline period. For instance, at follow-up all respondents had a regular place of care but only 21 had one at baseline, consequently the paired data *n* = 21. Where the paired sample size is *n* < 20, we report results descriptively.

#### Qualitative analysis

Patient responses in interviews were used to shed light on the causal pathways and to complement the quantitative findings. Inputs and outputs from the theory-based logic model guided the interview guide and initial coding for the qualitative analysis of interviews. Emergent codes were developed for statements about causal pathways in the logic model, such as statements about how the volunteer navigator influenced the patient’s first visit, or how the first visit influenced the patient’s access to health care. All interviews were French and French citations have been translated into English for publication.

## Results

A total of 8 volunteers were recruited and trained, of whom 5 were active for at least 4 weeks of the 17-week pilot intervention. New patients were assigned to two participating clinics where family doctors were accepting new patients. Over the 5-month trial period, 175 new patients were considered eligible for the telephone outreach intervention and 139 (79%) were contacted successfully and offered the welcome service, of which 108/139 (79%) accepted the service and 21% refused or said they did not need it.

Everyone who received the intervention was invited to participate in the evaluation but only 60/108 (55.6%) accepted and completed the baseline telephone-administered patient survey; 54/60 (85%) completed the three-month follow-up survey. Table 1 presents the sociodemographic and health profiles of the survey respondents and of those who participated in the individual interviews. Although we selected persons from neighbourhoods classified as socially-or-materially deprived, the profile of the study sample shows individual participants did not necessarily have low financial or educational status or low social support.

**Table 1:** Starting conditions: Description of patient sample for the patient survey and qualitative interviews

Characteristic	Study evaluation sample – completed the patient survey *n* = 60	Sub-sample with structured interviews (*n* = 13)
**Demographics**		
Mean age	49.3 years (SD = 13.9)	51.7 years (SD = 14.9)
Percent women	61.7% (37)	76.9% (10)
Percent unilingual Francophone	73.3% (44)	76.9% (10)
Percent Canadian-born	75% (45)	84.6% (11)
Percent Indigenous status	1.7% (1)	7.6% (1)
**Social characteristics**		
Self-perceived financial status	20.0% (2)	
Poor or tight	23.7% (14)	38.5% (5)
Modestly comfortable	50.8% (30)	15.4% (2)
Comfortable	5.1% (3)	46.2% (6)
Very comfortable		0% (0)
Highest educational achievement		
High school or less	31.7% (19)	38.5% (5)
Some post-secondary	68.3% (41)	61.5% (8)
Occupation		
Employed or in school	58.6% (34)	41.7% (5)
Looking for work	3.4% (2)	0% (0)
Not looking for work	37.9% (22)	58.3% (7)
Percent with low social support	13.8% (8)	15.4% (2)
**Health**		
Number of chronic conditions		
0	20.0% (12)	23.1% (3)
2-Jan	50.0% (30)	30.8% (4)
5-Mar	21.7% (13)	38.5% (5)
6 +	8.3% (5)	7.7% (1)
Perceived health		
Poor or fair	28.3% (17)	38.5% (5)
Good	43.3% (26)	30.8% (4)
Very good or excellent	28.3% (17)	30.8% (4)

Most respondents, 77% (46/60), reported needing medical care or advice in the previous 6 months, of whom the majority (59%, 27/46) reported difficulty getting care, leading to forgone care in most (74%, 20/27). Although these patients were on a waiting list for family doctors, at baseline 15% (9/60) reported having health professional responsible for most of their care. Of those without a most responsible professional, 41% (21/51) reported having a usual place of care.

In Fig. 2, the results of the evaluation are summarized by showing checks, where the data supports that the intended dimensions or causal pathway was achieved as hypothesized; “X” shows where dimensions or pathways were not achieved as hypothesized.

### Logic model activities and outputs: ability to perceive, seek, reach and engage

The key activity of the intervention was to inform the patients of the importance of attending the first visit (ability to perceive). We did not have indicators of ability to perceive in the survey, so findings are from the qualitative analysis. Most interviewed patients perceived the first visit as an administrative requirement to register and it came as a surprise that they would be returned to the waiting list if they did not attend. This awareness sharpened their intention to attend and to prepare for the visit.

*“Getting the call changed my perception of the first appointment with the doctor. Before it, I viewed such an appointment as a ‘first contact.’ Without the (volunteer navigator service), I don’t think I would have thought of mentioning my allergies, for example, or my sinus problems. A lot of the questions the volunteer mentioned good to ask, I considered banal, but I asked them anyway and they were actually quite helpful.” (Man, Age 31)*

Table 2 lists our findings on patient-reported changes in abilities to seek, reach and engage with health care. The pre-post measure of ability-to-seek improved statistically significantly from a mean of 2.7 to 3.3 (corresponding to less than moderately easy to very easy). The values increased especially for indicators in the construct on ease of finding needed healthcare, deciding which health professional, knowing about rights to services.

**Table 2:** Evidence of intended outputs of volunteer navigator activities: changes in patient abilities to access healthcare

Theoretical dimension	Box #, Logic model component	How measured	Baseline	3-month FU *n* = 54	Paired t-test (*n* = paired observations)
Ability to perceive	Box 1. Perceives need to attend PHC visit	Qualitative interview; Patient report at follow-up survey	---	---	---
Ability to seek	Box 2. Knows where and when to seek PHC	Survey: Ease of finding health information and identifying the most appropriate source of care.^a^ (4 items, range 1 − 4)	2.72 (0.79) (*n* = 59)	3.33 (0.55) (*n* = 51)	t = 4.47, *n* = 51, *p* = 0.000
Ability to reach	Box 3. Knows how to get to GP/FP	Survey: Ease of travelling to the clinic (1 item, range 1 − 4)	3.46 (0.96) *n* = 28	3.98 (0.14) *n* = 50	t = 2.63, *n* = 19, *p* = 0.02
Ability to engage	Box 4. Can engage with primary care	Survey: Ease of explaining needs to health professionals (1 item, range 1 − 4)	3.42 (0.77) *N* = 59	3.76 (0.56) *n* = 50	t = 3.26, *n* = 50, *p* = 0.002

*PHC* Primary Health Care, *GP/FP* General Practitioner or Family Physician

^a^1 = not at all easy, 2 = a little easy, 3 = easy, 4 = very easy

For ability to reach care, the volunteer navigators also gave practical transportation information. By design, patients on the wait list are allocated doctors within 5 km of their residence. The ease of travelling to the clinic showed statistically significant improvement.

Ability to engage with care also improved. Respondents’ ease of explaining their health problems to health professionals increased from an average of 3.4 at baseline to 3.8 at follow-up (corresponding to moderately to mostly very easy). Interviewed patients referred to the helpfulness of the volunteer navigator’s visit-preparation tips and pamphlet that had guided questions to clarify the patient’s reasons for the visit and help them organize their thoughts prior to and during the visit.

*“I remember using the pamphlet I received; I wrote down my questions. I’m certain I would have forgotten once at the clinic appointment.” (Man, Age 31)*

### Intended immediate outcome: first visit attendance and attachment

All 54 patients who participated in the follow-up survey presented to their first visit. (We do not have information on the 6 lost to follow-up or the 48 who received the service but did not participate in the evaluation.) Among participants who attended the first visit, only 89% (48/54) attached successfully; 3 had refused to register with the assigned family doctor and 3 did not answer affirmatively when asked if they had a most responsible doctor and were presumably undecided. Qualitative interviews with two undecided patients suggest attachment hesitancy after the first visit. One patient expressed irritation at the assigned doctor’s distraction with telephone calls during the consultation (Woman, Age 49). Another seemed torn between being grateful to have a family doctor and disappointed in not having her health problems taken seriously (Woman, Age 78).

The qualitative interviews suggest that patients went to their first visit predisposed to attach because most interviewed patients attributed the care shown in the telephone outreach by volunteer navigators to their new care teem and consequently they adopted a positive attitude to the first visit. Several interviewed patients said that the call gave them a sense that their new provider would take their care in hand and look after them.

*“For me, speaking with the (volunteer navigator) showed that there is good support and care (French: “prise-en-charge”) at this clinic, especially given that this was my first appointment with this new doctor.” (Man, Age 31)*

### Immediate impact: positive experience and enduring relationship

The visit with the new family doctor was generally experienced positively compared to the visit to the last professional seen at baseline. Table 3 shows demonstrate statistically significant improvements in patient-centered communication by the new doctor compared last baseline visit (*n* = 46) with a higher proportion of patients reporting that the new doctor had ‘completely’ discussed the most important issue, explained their health problem well, listened attentively and understood the patient, compared to the last visit at before being attached. Likewise, when asked whether they had confidence and trust in the provider seen in the last visit, 53% said ‘yes, definitely’ at baseline, compared to 77% at follow-up.

**Table 3:** Evidence of intended immediate impacts: Patient-reported changes visit experience and enduring relationship (Statistical testing only if > 20 paired observations)

**Intended impact:** **Box #, Logic model component**	**How measured**	**Baseline, in last 6 months** ***n* = 60**	**Follow-up, in last 3 months** ***n* = 54**	Test of difference* (*n* = paired observations)
Box 6. Healthcare is perceived positively	Discussed most important problem	47.7% (21/44)	69.2% (36/52)	*p* = 0.12, *n* = 36
Patient reports that at last visit doctor ‘completely’:	Explained problems well	38.5% (15/39)	60.0% (30/50)	*p* = 0.02, *n* = 30
	Seemed to understand the patient	43.2% (19/44)	73.1% (38/52)	*p* = 0.002, *n* = 36
	Listened attentively	42.2% (19/45)	80.8% (42/52)	*p* = 0.002, *n* = 37
	Patient ‘definitely’ has trust and confidence in health professional seen at last visit	53.3% (24/45)	77.4% (41/53)	*p* = 0.12, *n* = 38
Box 7. Enduring relationship with GP/FP/clinic	Patient feels known ‘very well’ as a person by regular doctor	8.3% (2/24)	4.4% (2/45)	n/a
	Regular doctor knows most important health concerns ‘very well’	26.1% (6/24)	48.8% (22/54)	n/a
	Patient feels ‘very comfortable’ discussing any health problem	47.8% (11/24)	85.4% (41/54)	n/a
	The provider knows ‘very well’ the patient’s health concerns	26.1% (6/24)	47.8% (22/54)	n/a

^*^McNemar test for binary variables and paired t-test for continuous variables

An enduring therapeutic primary care relationship was an intended impact, but indicators suggest that at 3 months, the new relationship was still in development. Asked how well they felt known as a person (including their values and beliefs), most of 24 respondents with a regular provider at baseline said ‘only a little’ (although 8.3% felt well known) and this improved slightly but not statistically significantly at follow-up among the 45 who responded about their new provider (only 4.4% felt well-known). Likewise, there is only a slight improvement in how well the provider was reported to know the patient’s health concerns. This is not surprising given that during the 3-month follow-up only 48% of respondents had more than one visit with the new doctor. Nonetheless, the foundations for an enduring relationship appear solid as reported comfort to discuss all their health problems with the doctor increased from 48% among the 24 with a regular provider at baseline to 85% among the 48 who registered with the new family doctor.

### Intended outcome: ongoing appropriate primary care

The achievement of ongoing appropriate care is predicated on the presumed clinical competence and responsiveness of the care team to give good quality, comprehensive, continuous and coordinated care. Although not measured specifically, our findings suggest enhanced patient access to ongoing appropriate primary care (Table 4).

**Table 4:** Intended Ultimate Outcome: Patient-reported indicators of ongoing appropriate primary care

**Intended outcome of appropriate care**	**Baseline, in last 6 months** ***n* = 60**	**Follow-up, in last 3 months** ***n* = 54**	**Paired t-Test of difference** (*n* = paired observations)
**Consumers’ needs addressed ‘in right location’ (Box 8)**			
Mean number of visits to a primary care doctor’s office or clinic (Standard Deviation)	1.2 (2.4)	2.00 (1.6)	*p* = 0.04 *n* = 54
Used community health or social services for a specific health problem	5.9% (3/51)	16.7% (8/48)	*p* = 0.13 *n* = 46
Regular doctor referred to a community health or social service	4.1% (1/24)	12.5% (6/48)	*p* = 0.06 *n* = 15
**Reduced forgone care and use of hospital emergency room**			
Experienced difficulty getting care among those needing care	53.5% (23/43)	18.4% (7/38)	*P* = 0.007 *n* = 36
Forgone care due to access difficulty	40.8% (20/49)	12.2% (6/50)	*p* = 0.013 *n* = 42
Use of the hospital emergency room	20.0% (12/60)	11.1% (6/54)	*p* = 0.29 *n* = 54
Among ER users, used due to difficulty accessing primary care	75.0% (9/12)	0% (0/6)	–

Several interviewed patients mentioned that they were referred to other providers or for diagnostic testing, suggesting increased comprehensiveness of care. Reported use of community health and social services for help with a specific health problem increased, from 6% (3/51) at baseline to 17% (8/48) at follow-up, suggesting increased appropriate referrals. Engaging with other care team members at the clinic, especially nurses, seemed to be critical to building the confidence of interviewed patients that they were in good hands and would get good care in the future.

Indicators of unmet needs suggest newly-attached patients reported more appropriate primary care compared to baseline. The rate of reported forgone care due to access difficulties dropped statistically significantly from 41% at baseline to 12% at follow-up. Self-reported emergency room use was 20% over the baseline 6 months and fell to 11% over 3 months’ follow-up. Although this suggests little overall change in rates, both quantitative and qualitative results suggest a meaningful change. From the survey, the self-reported reasons for emergency room use were very different at baseline than at follow-up. During baseline 9/12 (75%) emergency room visits included access difficulties (no family doctor or usual place of care, appointment wait too long); at follow-up, none of these reasons were reported, and the access-related reasons were to get timely diagnostic testing or specialist consults. Qualitative interviews suggest that information provided by the volunteer navigator contributed to the reduced emergency room use:

*“Before…, I would go to the hospital in case of urgent need. But since I got the call (from the volunteer), I know I can always call the clinic, and I’ll get an appointment almost immediately.” (Woman, Age 57)*

## Discussion

This mixed-method evaluation largely confirms the theory-based program logic model and suggests that volunteer navigators are a potentially promising innovation. In the patients willing to participate in the evaluation, the telephone outreach by volunteer navigators to unattached patients from socially-or-materially deprived neighbourhoods helped patients reach and engage successfully with newly-assigned family doctors, leading ultimately to the policy goal of more appropriate care for the population. Several intended consequences can be attributed directly to the intervention: improved care-seeking abilities; improved ability to explain health concerns to health professionals; and all patients who completed the follow-up survey presented for their first visit. The outreach by volunteer navigators predisposed patients to attach successfully to the newly-assigned family doctor. Most patients expressed high levels of confidence and trust in the family doctor and were in the process of establishing an enduring relationship. As a result of successful attachment more patients obtained referrals to other health and social services in the community, with dramatic reductions in the reported rates of access difficulties and forgone care.

Unlike clinic staff, volunteer navigators were able to reach out patients after office hours and take time to provide needed information. The act of outreach itself also seems to have contributed to the potential effectiveness by positively predisposing patients to the new care team. This act of pre-emptive care is particularly important for socially or materially deprived persons who expressed in our needs assessment that it is difficult to build trust with health professionals who live with very different social conditions than they do and that they often feel blamed for their health problems. Although not all persons reached by the navigator wanted or needed the navigation outreach, the qualitative findings suggest that even when patients did not think they needed the information or said they found the information self-evident during the call, they recognized its helpfulness when reflecting on it three months later.

Successful attachment for persons from socially-or-materially deprived neighbourhoods is significant because studies show that socioeconomically disadvantaged persons face challenges in attaching successfully to doctors [11, 28]. Patients from deprived neighbourhoods wait longer on the waiting list than their counterparts from advantaged neighbourhoods and coming from a socially-or-materially deprived neighbourhood or having a mental health diagnosis decreases the likelihood of attaching to a family doctor [13].

Interviews with family doctors prior to designing the intervention revealed a reluctance to accept new patients with social needs or mental health diagnoses that extended beyond their medical competence or comfort. This reluctance may translate into reduced efforts by clinic staff to attach new patients [29]. The volunteer navigators helped bridge the gap between the clinic and the community by making multiple contact attempts outside of office hours and informing patients about the consequences of not attending the first appointment, which came as a surprise to many. The fact that all those surveyed attended their first visit and that almost all enrolled formally with the assigned family doctor suggests that integrating patient navigators to the process of assigning patients to new doctors could reduce significantly the rate of persons being returned to the centralized waiting list.

Despite the modest sample size, we found a statistically significant improvement in patients knowing where and how to seek care and in ease of explaining their health problems to health professionals. This is significant because an analysis across all the IMPACT study regions shows that deprivation is correlated with lower abilities to access care and with higher rates of access difficulties, forgone care and use of emergency rooms [30]. The significant increases in abilities to seek, reach and engage with care are expected to translate into better ongoing access in the future, an effect we could not verify in this pilot study.

### Limitations

Although the findings generally support the effects and pathways proposed in our logic model, we recognize that these only demonstrate potential effectiveness, and that the results need to be interpreted with prudence despite statistical significance, because of the small sample size, lack of a comparison group and potential selection bias since only a little over half of those who received the service participated in the evaluation. We did not adapt the critical values of our statistical testing for multiple testing, but we reduced type 1 errors by limiting our testing to hypotheses stated à priori as per the logic model. For instance, we found that self-perceived measures of health improved statistically significantly over follow-up, but we did not report this because we had not hypothesized such an impact with the intervention. Data collection was driven by the IMPACT program logic model for the whole, so we may have missed or obtained limited information on key domains or pathways specific to this intervention. The qualitative data provided critical insights where quantitative data were uninformative. For instance, we cannot claim that the intervention reduced emergency room use because of our small sample size and possible time-frame overlap but the qualitative interviews and elicited reasons for use are consistent with a meaningful change. Despite the limitations, the formulation of a theory-based logic model and mixed-method data collection enabled exploration of causal mechanisms as well as unintended effects, such as reasons for not attaching to the assigned family doctor despite the navigator help.

Despite the intent to target socially-or-materially deprived persons, in our study sample only 12% had high deprivation defined as two or more of the following: low education, poor financial status, limited language proficiency, indigenous status or low social support. It was 19% in other IMPACT regions. This is perhaps not surprising because area-based socioeconomic indicators are less sensitive to health inequalities than are individual-level indicators [31]. Also, enrollment on the centralized waiting list is lower-than-expected for persons from deprived neighbourhoods, [13] and it is probable that even within these neighbourhoods the more deprived persons are less likely to enroll. This demonstrates the challenge of appropriately targeting programs to vulnerable populations. Nonetheless, 35.6% of the study sample reported it being not easy to find health and healthcare information. The proactive outreach by volunteer navigators would be critical for this group of persons with low health information agency.

## Conclusions

We are reasonably confident in asserting that telephone outreach by volunteer navigators helps patients from socially-or-materially deprived neighbourhoods attach successfully to newly-assigned family doctors and have a positive healthcare experience. As per the logic model, successful attachment led to the achievement of the intended policy goals of helping the population get more appropriate primary care, reduce rates of forgone care and change use of hospital emergency rooms. This volunteer-based intervention allowed the introduction of a service delivery despite a context of budget cuts and structural changes in the health system. Our health system partners decided to sustain this light-touch navigational model, and we are currently exploring sustainability and spread to new contexts. Trained lay navigators by their social proximity to deprived patients may help bridge gaps in primary healthcare delivery and have the potential to improve equity of access to primary healthcare.

### Supplementary Information

**Additional file 1.**

## Data Availability

The datasets analysed during the current study are available from the corresponding author on reasonable request, as are detailed reports of the qualitative and quantitative analyses.

## Abbreviations

GP/FP: General Practitioner or Family Physician
IMPACT: Innovative Models Promoting Access-to-Care Transformation
PHC: Primary Health Care
