# Supplementary material for: Telephone outreach by volunteer navigators: a theory-based evaluation of an intervention to improve access to appropriate primary care
Source: BMC Prim Care. 2023 Aug 21;24:161. doi: 10.1186/s12875-023-02096-4 (PMC10441746; doi:10.1186/s12875-023-02096-4)
Supplement: Supplementary file 1 — Additional file 1. [file 12875_2023_2096_MOESM1_ESM.docx]

My first questions will allow us to assess how the newly offered service could be beneficial for you and for the health system. There are no right or wrong answer. Most of them will be on your experience of care but some will be a bit more personal. Let start…

**What is your date of birth?**

**Your postal code?**

**Are you?**

**☐** Male

**☐** Female

**☐** Other, please specify:

**What language(s) do you usually speak at home?** *Check all that apply*

- English
- French
- Another language, please specify: ______________

**Were you born in Canada?**

**☐** Yes

**☐** No

**If no, in what year did you first come to Canada to live?**

**Are you aboriginal?**

**☐** Yes

**☐** No

**Which of the following best describes the highest level of education you have completed?**

- *Don’t read/Select the highest level*
-  Completed a graduate or professional degree (Master, MD, DDS, DMD, DVM, OD, PhD)
-  Completed a bachelor’s degree (e.g. B.A., B.Sc.,B.S.N.)
-  Had some university education or completed a community college, technical college, or postsecondary program (e.g. trade, technical or vocational school, CEGEP)
-  Completed secondary school or high school
-  Did not complete secondary school or high school

These next questions are about health services you have used or needed in the last 6 months, that is since [month, year]

**In the past 6 months, did you need any health services or medical advice?**

☐ No

☐ Yes

**In the last 6 months, where did you go to receive care?**

*Don’t read options ; check all that apply*

□ No where, I did not consult about my health over the last 6 months

□ At my usual Clinic

□ In only one clinic or CLSC (other than my usual clinic)

□ More than one clinic or CLSC

□ Only one specialty clinic

□ More than one specialty clinic

□ An outpatient clinic in a hospital

□ An emergency room of a hospital

□ In another place, specify:________________________________

**In the past 6 months, were there times when you had difficulty getting the healthcare or advice you needed** (You had to make several attempts, call or go to different places)**?**

☐ N/A, I didn’t need any healthcare or advice in the last 6 months

☐ No → *Go ask q.X*

☐ Yes, once

☐ Yes, several times

*→ If YES to the above question, ask the following without reading the list of answers and check all that apply. Prompt by offering categories:* Problem contacting a doctor or a place, Time delay, Language, transport or cost problem, personal reason? Leave a bit of time after mentioning each category.

**What type of difficulties did you experience?** (These include all difficulties associated with your condition, to health issues or to the health system)

Contact ☐ Difficulty contacting a doctor

☐ Difficulty getting an appointment

☐ Did not know where to go

☐ I do not have a doctor or a nurse

☐ A specialist was not available

Time/delay ☐ Too long a wait to get an appointment

☐ Too long a wait in the waiting room

☐ Service not available at the time required

☐ Service not available in that area

Barriers ☐ Transportation problems

☐ Unable to leave the house because of a health problem

☐ Cost issues

☐ Language barriers

Personal Issues ☐ I didn’t think anything would help

☐ I was afraid to ask of help or what others would think of me

☐ I asked but didn’t get help

☐ Did not feel comfortable with the available doctor or nurse

☐ Other, please specify: ____________________________________

**Did any of the difficulties STOP you from getting healthcare or advice needed?**

☐ No

☐ Yes**When was your last visit to a doctor’s office, a clinic, a walk-in clinic or a CLSC?**

☐ Less than 6 months ago

☐ Between 6 and 12 months ago

☐ Between 1 and 2 years ago

What type of place was it? *Make sure it is really a primary care setting*

**Who did you see?**

☐ My doctor

☐ Another doctor or nurse in my usual place of care

☐ Another doctor or nurse in another setting

☐ Other, please specify: (*If this option is chosen, make sure it was in a doctor’s office, walk-in clinic or CLSC*)

**In the past 6 months, how many visits have you had with a doctor or nurse in an office setting, in your own home or at a walk-in clinic (not including specialists like cardiologist, oncologist, etc.)?**  Visits

**Have you seen more than one doctor or nurse in the past 6 months (not counting those seen during hospitalizations or specialists)?**

☐ No

☐ No, only 1

☐ Yes, 2 or 3

☐ Yes, 4 or more

Now, were going to ask you a series of questions about health services you may have used in the past 6 months, since…(repeat month)

**In the past 6 months, how many visits have you had to an emergency department?**

*If more than 1 visit, if 0 don’t ask the next two questions*

**Did you use the emergency department because of the difficulties in getting health care or advice you needed?**

☐ No

☐ Yes

**Could you tell me what were the reasons for choosing to go to the emergency room?**

*Don’t read, check as many as apply*

☐Regular doctor not available

☐Because nobody was available at your regular clinic

☐Do not have a regular doctor or clinic

☐Required services not available at the clinic

☐Transportation problem to the clinic/too far (easier to go to the Hospital)

☐Higher cost to get to the clinic

☐Difficulty getting an appointment at the clinic

☐Next appointment too far/delay too long

☐Wait time in the waiting room too long at the clinic

☐Didn’t know where to go, lack of information about available services

☐Was told to go to the ER (info santé; 811; etc.)

☐Not sure to be able to receive healthcare at my clinic/opening hours, walk-in availability

☐Clinic not open when could attend

☐Has an appointment but has not seen the doctor yet (appointment to come but immediate need)

☐Need to see a specialist in a reasonable time

☐Need to see have tests done in a reasonable time

☐When things went wrong or changed I could not get answers or advice quickly

☐Best place to have my problem taken care of

☐Did not have the information I needed to cope with my health

☐ Did not feel comfortable with the doctor or nurse I saw

☐ I asked but did not receive help

☐ Other, specify:

**The next three questions are about support from your family and friends.**

I**f you needed it, how many persons, family or friends, could freely help you with activities of daily living (e.g. dressing, driving)?** *Read options*

☐ More than one

☐ One

☐ None

**How many persons, family or friends, show you love and affection when you need it?**

☐ More than one

☐ One

☐ None

**With how many persons, family or friends, can you freely confide in or talk about yourself or your problems?**

☐ More than one

☐ One

☐ None

**What phrase best describes your financial situation?**

*Read options*

 Very comfortable

 Comfortable

 Modestly comfortable

 Tight

 Very tight

 Poor

Calculate the Social Vulnerability Index with all 6 items identified in blue-green

If les than 2 – Thank you and end interview

If equal or more than 2 – Say eligible for intervention, ask for verbal consent, ask to continue interview

Before starting the section of the survey about your healthcare experience, I would like to tell you what we would like you to think about. We would like you to refer to the person who takes care of your healthcare in general in a doctor’s office, a clinic, a CLSC or a FMG; but not in a hospital. So, thinking about these places…

**Is there a specific health professional who is mainly responsible for your healthcare?**

☐ No → *Go to question nest question*

☐ Yes

**If yes, what type of health professional is he/she?**

☐Family [doctor] or general practitioner

☐Nurse/Nurse Practitioner

☐Social worker

☐Specialist (cardiologist, internist, etc.)

☐Chiropractor, acupuncturist, naturopath or other alternative medicine

**How many years have you had this doctor?** Years

**Would you recommend your doctor to a friend or relative?**

☐ No

☐ Yes

**Is there a specific place that you usually go when you are sick or need advice about your health?**

☐ No → *Go to the next section*

☐ Yes

Thinking about the clinic where you usually see your doctor OR usually go to for your healthcare

**Is it easy for you to reach this place by phone?**

☐Very easy

☐Moderately

☐A little

☐No, not at all

**Is it easy for you to get medical advice at this place by phone?**

☐Very easy

☐Moderately

☐A little

☐No, not at all

**Is it easy for you to travel to this place?**

☐Very easy

☐Moderately

☐A little

☐No, not at all

**At your clinic, how helpful do you find the office staff?**

☐Very helpful

☐Moderately helpful

☐Somewhat helpful

☐Not at all helpful

The healthcare team is composed of all doctors (family doctors and specialists), nurses and other health professionals who you see at your clinic

**At your clinic, other than the person who takes care of most of your healthcare, how much can you count on everyone on the care team for help?**

☐ A lot

☐ Moderately

☐ Somewhat

☐ Not at all

**In the last 6 months, WHO did you consult at your Clinic?**

*Check as many as apply*

□ My usual doctor

□ Another family doctor or general practitioner

□ A specialist

□ A nurse

□ A nutritionist or a dietician

□ A physiotherapist or an occupational therapist

□ A psychologist or a social worker

□ Other, please specify: ____________________________________

□ I did not go to my Clinic over the last 6 months

**Other than your Clinic, WHO did you consult in the last 6 months?**

*Check as many as apply*

□ A family doctor or general practitioner

□ A specialist

□ A nurse

□ A nutritionist or a dietician

□ A physiotherapist or an occupational therapist

□ A psychologist or a social worker

□ Other, please specify: ____________________________________

□ No one other than my Clinic

**How much do staff at the clinic help you get the services you need from other places** (like places you have to go to get specialized care, specific exam, counselling or physiotherapy)**?**

☐ A lot

☐ Moderately

☐ A little

☐ Hardly at all

Thinking about the difficulty of getting care you need, in the last 6 months,

**How often did you NOT take drugs that were prescribed by a doctor or nurse because of their cost?**

☐ Never OR rarely

☐ Sometimes

☐ Often OR very often

**How often did you NOT take laboratory tests or exams that were prescribed by a doctor or nurse because of their cost** (like blood draw, X-rays, etc.)**?**

☐ Never OR rarely

☐ Sometimes

☐ Often OR very often

**How often did you decide NOT to get services you because of costs like transportation, babysitting, or losing pay from work?**

☐ Never OR rarely

☐ Sometimes

☐ Often OR very often

**Over the past 6 months, were there times during or between healthcare visits when you felt abandoned or left too much to your own resources?**

☐ No, never → *skip the next question*

☐Yes, at times

☐Yes, often

☐Yes, always

**What were the reasons?** *Don’t read, check as many as apply*

☐ Do not have a regular doctor or clinic

☐ Regular doctor not available

☐ Too difficult or too long to be seen at my regular clinic

☐ Too difficult or too long to be seen by a specialist or another person that I had been referred to

☐ Person saw didn’t really know my personal health situation

☐ Person saw didn’t really know my medical history

☐ No one seemed to be in charge of my healthcare

☐ Person saw didn’t seem to know about what others had done or told me

☐ Didn’t know what were the next steps in my care

☐ When things went wrong or changed I could not get answers or advice quickly

☐ Persons I saw gave me different information

☐ Didn’t have the information needed to cope with health between appointments

☐ Not comfortable with my doctor/do not trust my doctor or nurse

☐ I didn’t feel understood

☐ Other, specify:

**In the past 6 months, have you missed an appointment with your doctor?**

☐ No, never

☐ Yes, only once

☐ Yes, 2 to 3 times

☐ Yes, more than 4 times

**In the past 6 months, counting overnight stays (if any), what is the total number of nights that you have spent in:**

a. a hospital? __

b. a long term care home/facility? __

**In the past 6 months, have you consulted a health professional about your emotional or psychological health?**

□ No

□ Yes

**Whom did you see or talk to?**

*Check as many as apply*

□ Family doctor or general practitioner

□ Psychiatrist

□ Psychologist

□ Nurse

□ Social worker or counsellor

□ Other – Specify: ______________________________

These next questions are about health or social services you might have received in the community. These are health-related services like nutritionists, exercise support, dental care, psychologist, self-help groups, or home care.

**Has your doctor ever recommended a health or social service in the community for a specific**

**health problem?**

☐ No

☐ Yes

**Did someone else recommend you to those services?**

☐ No

☐ Yes

**In the last 6 months, have you used** **a health or social service in the community for a specific**

**health problem?**

☐ No

☐ Yes

**Which one?**

**In the last 6 months, have you seen or talked to a health professional about specific things to improve your health or prevent illness (such as smoking cessation, limiting alcohol drinking, exercise, etc.)?**

☐No

☐Yes

**Has your doctor or nurse provided everything you need to help you manage your health concerns?**

☐Yes, definitely

☐Yes, to some extent

☐No, not really

☐No, not at all

**In the last 6 months, were you encouraged to go to a specific group or class to help you manage your health concerns?**

☐Yes, definitely

☐Yes, to some extent

☐No, not really

☐No, not at all

☐No, I haven’t needed such support

**Has support from the local health and social services helped you manage your health concerns (**Excluding family GP/doctor’s services)**?**

☐Yes, definitely

☐Yes, to some extent

☐No, not really

☐No, not at all

☐No, I haven’t needed such support

**In the past 6 months, have you called a telephone help line or 811 for medical or health information or advice?**

☐No, never

☐Yes, sometimes

☐Yes, often

For the next questions, think about this person you saw on that last visit…

**To what extent was the problem that you consider most important discussed?**

☐Completely

☐Mostly

☐A little

☐Not at all

**To what extent did the doctor or nurse explain your problem to you?**

☐Completely

☐Mostly

☐A little

☐Not at all

**How well do you think the doctor or nurse understood you during this visit?**

☐Completely

☐Mostly

☐A little

☐Not at all

**To what extent did the doctor or nurse listen to you carefully?**

☐Completely

☐Mostly

☐A little

☐No, not at all

**To what extent did the doctor or nurse show respect for what you had to say?**

☐Completely

☐Mostly

☐A little

☐No, not at all

**Did the doctor or nurse spend enough time with you?**

☐ Yes, absolutely

☐Mostly

☐A little

☐No, not at all

**Did you have confidence and trust in the person you saw or spoke to?**

☐Yes, definitely

☐Yes, to some extent

☐No, not at all

☐Don’t know/can’t say

☐ I did not see a family physician or nurse

These next questions are about your doctor or nurse mainly responsible for your healthcare and identified earlier. In general…

**How well would you say that your doctor or nurse knows what worries you most about your health?**

☐ Very much

☐ A fair amount

☐ A little

☐ Not at all

**How well would you say that your doctor knows you as a person? (your values, beliefs)**

☐ Very much

☐ A fair amount

☐ A little

☐ Not at all

Now, over the last 6 months, since…

**How much importance does your doctor or nurse give to your ideas about your care?**

☐Great importance

☐Moderate importance

☐Little Importance

☐Hardly any importance

**How comfortable do you feel discussing with your doctor or nurse personal problems related to your health condition?**

☐Very comfortable

☐Moderately

☐Only somewhat

☐Not very comfortable

**How confident are you that your doctor or nurse will look after you no matter what happens with your health?**

☐Very confident

☐Moderately

☐Only somewhat

☐Not very confident

Thinking about the times you were recommended to go to a program in the community for a specific health problem.

**Now, thinking specifically about your doctor or nurse, how much does your doctor or nurse help you get the health care you need from other places?**

☐ A lot

☐ Moderately

☐ A little

☐ Hardly at all

**How much help did your doctor or nurse give you in deciding who to see?**

☐ A lot of help

☐ Moderately

☐ A little

☐ Not at all

**How much does your doctor or nurse or nurse keep in contact with you even when you receive healthcare in other places?**

☐ A lot

☐ Moderately

☐ A little

☐ Not at all

Thinking about you

**In general, how easy is it for you to get health information by yourself when you need it?**

☐Very easy

☐Moderately easy

☐Not very easy

☐Not easy at all

**How easy is it for you to find out which health services you have the right to receive?**

☐Very easy

☐Moderately easy

☐Not very easy

☐Not easy at all

**How easy is it for you to find the right place to receive the healthcare you need?**

☐Very easy

☐Moderately easy

☐Not very easy

☐Not easy at all

**How easy is it for you to decide which health professionals you need to see?**

☐Very easy

☐Moderately easy

☐Not very easy

☐Not easy at all

**How easy is it for you to explain your problems to health professionals?**

☐Very easy

☐Moderately easy

☐Not very easy

☐Not easy at all

**How easy is it for to take action to improve your health?**

☐Very easy

☐Moderately easy

☐Not very easy

☐Not easy at all

**Do you know the community and social organisations in your area?**

☐ Yes, a lot

☐ Yes, to some extent

☐ No, not really

☐ No, not at all

**Do you know the services they offer?**

☐ Yes, definitely

☐ Yes, to some extent

☐ No, not really

☐ No, not at all

**Have you ever used one of them?**

☐ Yes, definitely

☐ Yes, to some extent

☐ No, not really

☐ No, not at all

**Do you know how these services could help you manage your health?**

☐ Yes, definitely

☐ Yes, to some extent

☐ No, not really

☐ No, not at all

These next questions are about your health

**In general, would you say your health is:**

☐ Excellent

☐ Very good

☐ Good

☐ Fair

☐ Poor

The following questions are about activities you might do during a typical day.

**Does your health now limit you in moderate activities**, such as moving a table, pushing a vacuum cleaner, bowling or playing golf?

☐ Yes, limited a lot

☐ Yes, limited a little

☐ No, not limited at all

**Does your health now limit you in** climbing **several** flights of stairs**?**

☐ Yes, limited a lot

☐ Yes, limited a little

☐ No, not limited at all

For these next questions, think about the last four weeks, [since date]

**During the past four (4) weeks, have you accomplished less than you would like with your work or other regular daily activities as a result of your physical health?**

☐ No, none of the time

☐ Yes, a little of the time

☐ Yes, some of the time

☐ Yes, most of the time

☐ Yes, all of the time

**During the past four (4) weeks, were limited in the kind of work or other regular daily activities as a result of your physical health?**

☐ No, none of the time

☐ Yes, a little of the time

☐ Yes, some of the time

☐ Yes, most of the time

☐ Yes, all of the time

**During the past four (4) weeks, have you accomplished less than you would like** **with your work or other regular daily activities as a result of any emotional problems (such as feeling depressed or anxious)?**

☐ No, none of the time

☐ Yes, a little of the time

☐ Yes, some of the time

☐ Yes, most of the time

☐ Yes, all of the time

**During the past four (4) weeks, have you not done** **work or other activities as** **carefully as usual as a result of any emotional problems (such as feeling depressed or anxious)?**

☐ No, none of the time

☐ Yes, a little of the time

☐ Yes, some of the time

☐ Yes, most of the time

☐ Yes, all of the time

**During the past four (4) weeks, how much did pain interfere with your normal work (including both work outside the home and housework)?**

☐ Not at all

☐ A little bit

☐ Moderately

☐ Quite a bit

☐ Extremely

**These questions are about how you feel and how things have been with you during the past four (4) weeks. For each question, please give the one answer that comes closest to the way you have been feeling.**

**How much of the time during the past four (4) weeks have you felt calm and peaceful?**

☐ All of the time

☐ Most of the time

☐ A good bit of the time

☐ Some of the time

☐ A little of the time

☐ None of the time

**How much of the time during the past four (4) weeks did you have a lot of energy?**

☐ All of the time

☐ Most of the time

☐ A good bit of the time

☐ Some of the time

☐ A little of the time

☐ None of the time

**How much of the time during the past four (4) weeks have you felt downhearted and blue?**

☐ All of the time

☐ Most of the time

☐ A good bit of the time

☐ Some of the time

☐ A little of the time

☐ None of the time

**During the past four (4) weeks, how much of the time has your physical health or emotional problems interfered with your social activities (like visiting with friends, relatives, etc.)?**

☐ All of the time

☐ Most of the time

☐ Some of the time

☐ A little of the time

☐ None of the time

Now, we’d like to ask you some questions about how your health may have changed over the last 12 months.

**Compared to one year ago, how would you rate your physical health in general now?**

☐ Much better

☐ Slightly better

☐ About the same

☐ Slightly worse

☐ Much worse

**Compared to one year ago, how would you rate your emotional problems (such as feeling anxious, depressed or irritable) now?**

☐ Much better

☐ Slightly better

☐ About the same

☐ Slightly worse

☐ Much worse

**What is your height?**

**What is your weight?**

The next questions are about chronic health condition you might have.

**I’m going to read to you a list of conditions, tell me which one have lasted for more than 6 months or for which you are taking prescribed medicines, even if this problem is under control right now.**

*(Check “yes” only for conditions that have lasted for more than 6 months and have been confirmed by a doctor or nurse or for which prescribed medicines is taken)*

| **Yes** |  |
| --- | --- |
| ☐ | High blood pressure (Hypertension) |
| ☐ | High Cholesterol (Hyperlipidemia) |
| ☐ | Arthritis (Rheumatoid or osteoarthritis) |
| ☐ | Persistent back/sciatic pain or persistent joint and muscle pain (non-arthritis) |
| ☐ | Stomach problem (Reflux, ulcer or heartburn) |
| ☐ | Depression or anxiety |
| ☐ | Thyroid disorder |
| ☐ | Asthma, chronic obstructive pulmonary disease (COPD) or chronic bronchitis |
| ☐ | Diabetes |
| ☐ | Heart problem (angina, myocardial infarction, atrial fibrillation, poor circulation in lower limb) |
| ☐ | Bowel/Colon problem (irritable bowel, Crohn’s disease, ulcerative colitis, diverticulosis) |
| ☐ | None of the above mentioned |

**Is there anything else?** *If “Yes” the following table will appear. Don’t read, check all that was confirmed by a doctor or for which the respondent is taking medication*

| **Yes** |  |
| --- | --- |
| ☐ | Any cancer in the previous 5 years |
| ☐ | Liver problem (Chronic hepatitis) |
| ☐ | Osteoporosis |
| ☐ | Kidney disease or failure |
| ☐ | Chronic urinary problem |
| ☐ | Heart failure |
| ☐ | HIV/AIDS |
| ☐ | Stroke (including TIA - transient ischemic attacks) |
| ☐ | Dementia or Alzheimer’s disease |
| ☐ | Other, specify |
| ☐ | Other, specify |

For the next set of questions, you will have to provide an answer on a scale of 1 to 10, where 1 is “not confident at all” and 10 is “totally confident”. You can also tell me if the question does not apply to your situation. Thinking about your chronic condition(s) we just identified together…

**How confident are you that you can keep symptoms** (like fatigue, pain, discomfort, or emotional distress) **caused by your disease from interfering with things you want to do?**

☐ ☐ ☐ ☐ ☐ ☐ ☐ ☐ ☐ ☐

1 2 3 4 5 6 7 8 9 10

Not confident at all Totally confident

**How confident are you that you can do the different tasks and activities needed to manage your**

**health condition?**

☐ ☐ ☐ ☐ ☐ ☐ ☐ ☐ ☐ ☐

1 2 3 4 5 6 7 8 9 10

Not confident at all Totally confident

**How confident are you that you can do things other than just taking medication to reduce how much your illness affects your everyday life?**

☐ ☐ ☐ ☐ ☐ ☐ ☐ ☐ ☐ ☐

1 2 3 4 5 6 7 8 9 10

Not confident at all Totally confident

*If no usual source of care, skip this next section*

Thinking about the care you received over the past 6 months,

**Has your family doctor or nurse proposed a treatment plan for you?** (A treatment plan is a set of actions your family doctor or nurse recommends to manage your health problem)

☐Yes, definitely

☐Yes, to some extent

☐No, not really

☐No, not at all

☐ I haven’t needed a treatment plan

**Did your doctor or nurse give you a sense of control over your health?**

☐Yes, definitely

☐Yes, to some extent

☐No, not really

☐No, not at all

**Did your doctor or nurse help you feel that sticking with your treatment would make a difference?**

☐Yes, definitely

☐Yes, to some extent

☐No, not really

☐No, not at all

**Did your doctor or nurse help you feel confident about your ability to take care of your health?**

☐Yes, definitely

☐Yes, to some extent

☐No, not really

☐No, not at all

**What racial or cultural groups do you belong to?**

*Check all that apply* *; Don’t read but you may prompt*

☐ White (Caucasian/European)

☐ Aboriginal First Nation Status

☐ Aboriginal First Nation Non-Status

☐ Aboriginal Metis

☐ Aboriginal Inuit

☐ South Asian (e.g., East Indian, Pakistani, Sri Lankan)

☐ Chinese

☐ Black

☐ Filipino

☐ Latin American

☐ Arab

☐ Southeast Asian (e.g., Vietnamese, Cambodian, Malaysian, Laotian)

☐ West Asian (e.g., Iranian, Afghan)

☐ Korean

☐ Japanese

☐ Other group – Please specify:

**What is your current occupation?**

*(if more than one of these applies to you, please check the main ONE only)*

☐ Employed full time (including self-employed or on a work training program; 30 hours or more each week)

☐ Employed part time (including self-employed or on a work training program; under 30 hours each week)

☐ Unemployed and looking for work

☐ At school or in a full-time education

☐ Unable to work due to a long-term sickness or disability

☐ Looking after your home/family

☐ Retired from paid work

☐ Doing something else
